# Non-invasive pulsed ultrasound enhances hematoma clearance and neurological recovery in experimental intracerebral hemorrhage

**DOI:** 10.3389/fneur.2026.1698217

**Published:** 2026-02-11

**Authors:** Feifei Ma, Linlin Wang, Jinjin Wang, Yang Du, Wenyu Dong, Yanfang Li, Qixuan Guan, Wenrui Xing, Xiping Gong, Pei Dong, Li Guo, Ruijun Ji

**Affiliations:** 1 Department of Neurology, Beijing Tiantan Hospital, Capital Medical University, Beijing, China; 2 Department of Neurology, Peking University Sixth Hospital, Peking University Institute of Mental Health, Beijing, China; 3 China National Clinical Research Center for Neurological Diseases, Beijing, China; 4 Beijing Key Laboratory of Brain Function Reconstruction, Beijing, China

**Keywords:** intracerebral hemorrhage, non-invasive pulsed ultrasound, hematoma clearance, perihematomal edema resorption, neurological recovery

## Abstract

**Background:**

Intracerebral Hemorrhage (ICH) is a devastating stroke subtype. Accelerating hematoma clearance is a critical therapeutic goal. This study evaluated non-invasive pulsed ultrasound for enhancing hematoma clearance, edema resolution, and recovery post-ICH.

**Methods:**

Twenty-nine rats with striatal autologous blood-induced ICH were randomized into control (*n* = 11), 2 MHz ultrasound (*n* = 7), and 8 MHz ultrasound (*n* = 11) groups. Ultrasound treatment (60 min/day) was applied for 7 consecutive days following ICH induction. Hematoma volume and perihematomal edema (PHE) were assessed by T2-weighted imaging (T2WI) and susceptibility-weighted imaging (SWI) at days 1 and 7 post-ICH. Neurological function was assessed by corner turn and cylinder tests at baseline and days 1, 3, and 7.

**Results:**

Non-invasive pulsed ultrasound significantly enhanced hematoma clearance (2 MHz: 47.7%; 8 MHz: 47.8% vs. control: 20.4%, *p* < 0.01) and PHE resolution (2 MHz: 53.9%; 8 MHz: 71.8% vs. control: 31.1%, *p* < 0.05). Behavioral tests showed reduced right-turn bias and forelimb asymmetry in ultrasound groups (*p* < 0.05). No frequency difference was found.

**Conclusion:**

Non-invasive pulsed ultrasound significantly enhances hematoma clearance, reduces edema, and improves functional recovery post-ICH, supporting its translational potential.

## Introduction

Intracerebral hemorrhage (ICH) is a particularly devastating subtype of stroke, characterized by high morbidity and mortality, with limited effective therapeutic options (1). Although ICH accounts for only 10–25% of all strokes globally (2, 3), its 30-day mortality rate remains as high as 50% (4–6). The dual-injury paradigm, consisting of primary mechanical disruption and secondary neurotoxicity from hemoglobin degradation products, drives progressive neurological deterioration (7). The increase of 1 mL in absolute ICH volume leads to a 7% greater likelihood for patients to shift from independence to dependence (8).

Current management strategies for ICH remain largely supportive, focusing on blood pressure control, hematoma evacuation, and reducing secondary injury (9). However, clinical trials, investigating surgical hematoma removal, such as STICH (10), MISTIE (11), and CLEAR (12), have failed to demonstrate significant survival benefits. Notably, the MISTIE-III trial which aimed to refine minimally invasive techniques, also showed no overall improvement in functional outcomes compared to standard medical care, despite achieving hematoma volume reduction (13). Furthermore, the recent ENRICH trial revealed a critical nuance: minimally invasive evacuation significantly improved functional recovery in patients with lobar hemorrhages, yet demonstrated no efficacy for those with basal ganglia hemorrhages (14). As a result, alternative non-invasive therapeutic strategies are being actively explored to enhance hematoma clearance and promote neurological recovery.

Emerging as a promising non-invasive modality, ultrasound has recently garnered significant attention for its neuroprotective potential across multiple central nervous system disorders. Notably, preclinical studies have demonstrated its therapeutic utility in ischemic stroke (15), Parkinson’s disease (16), and traumatic brain injury (17). Low-intensity focused ultrasound reduces immunoglobulin G (IgG) deposition in the brain, indicating decreased blood–brain barrier (BBB) disruption, thereby contributing to neuroprotection following ischemic stroke (15). Similarly, in TBI, low-intensity pulsed ultrasound (LIPUS) significantly attenuates neuroinflammation by decreasing MMP-9 activity, neutrophil infiltration, and microglial activation (17). Moreover, studies suggest that ultrasound, through its cavitation and mechanical effects, can effectively fragment thrombi and calcified plaques into micron-sized particles, thereby facilitating their clearance (18, 19). In addition, continuous monitoring with 2-MHz transcranial Doppler (TCD) during tissue plasminogen activator (tPA) infusion for ischemic stroke has been associated with a high rate of complete recanalization and significant clinical recovery, likely by enhancing thrombolysis through increased clot surface exposure to tPA (20). Despite these promising findings, it remains unclear whether treatment with non-invasive pulsed ultrasound can alleviate ICH-induced detrimental outcomes and inflammatory responses.

In this study, we investigated the effects of non-invasive pulsed ultrasound (2 MHz and 8 MHz) on hematoma clearance, perihematomal edema (PHE) absorption, and neurological recovery in a rodent model of ICH. The 2 MHz frequency was chosen because it corresponds to the standard operating frequency of clinical transcranial Doppler systems and has well-established safety, skull penetration characteristics, and translational relevance. In contrast, 8 MHz ultrasound was included as a higher-frequency comparator to investigate whether ultrasound-mediated hematoma clearance and perihematomal edema resolution exhibit frequency-dependent characteristics.

We hypothesized that ultrasound stimulation would enhance hematoma resolution and attenuate secondary brain injury, thus improving functional outcomes. Our findings provide insight into the potential translational applications of non-invasive pulsed ultrasound as a novel therapeutic strategy for ICH management.

## Methods

### Animal model of intracerebral hemorrhage

All animal procedure protocols were approved by the Ethics Committee of the Beijing Neurosurgery Research Institute. The study complies with the ARRIVE guidelines for reporting *in vivo* experiments. Twenty-nine adult male Sprague–Dawley rats (250–350 g, Beijing Vital River Laboratory Animal Technology Co., Ltd.) were used. The rats had free access to food and water before and after surgery and were housed in a 12-h light/dark cycle. The animals were anesthetized with intraperitoneal injection of pentobarbital (45 mg/kg), and body temperature was maintained at 37 °C using a feedback-controlled heating pad. The rats were placed in a stereotactic frame (RWD, Shenzhen, China) and a cranial burr hole (1 mm) was drilled on the right coronal suture 3.5 mm lateral to the midline. Autologous arterial blood was obtained from the right femoral artery cannulation with a polyethylene catheter and injected immediately after collection, at a rate of 6 μL/min using a 26-gage needle at the coordinates: 0.2 mm anterior, 5.5 mm lateral, and 3.5 mm ventral to the bregma. Blood withdrawal and intracerebral injection were performed consecutively without storage or intentional delay, within a short and consistent time window, to minimize the possibility of *ex vivo* clot formation prior to injection. The needle remained in position for an additional 10 min before being gently removed. The burr hole was filled with bone wax, and the skin incision was sutured closed.

### Treatment allocation

**Figure 1:**
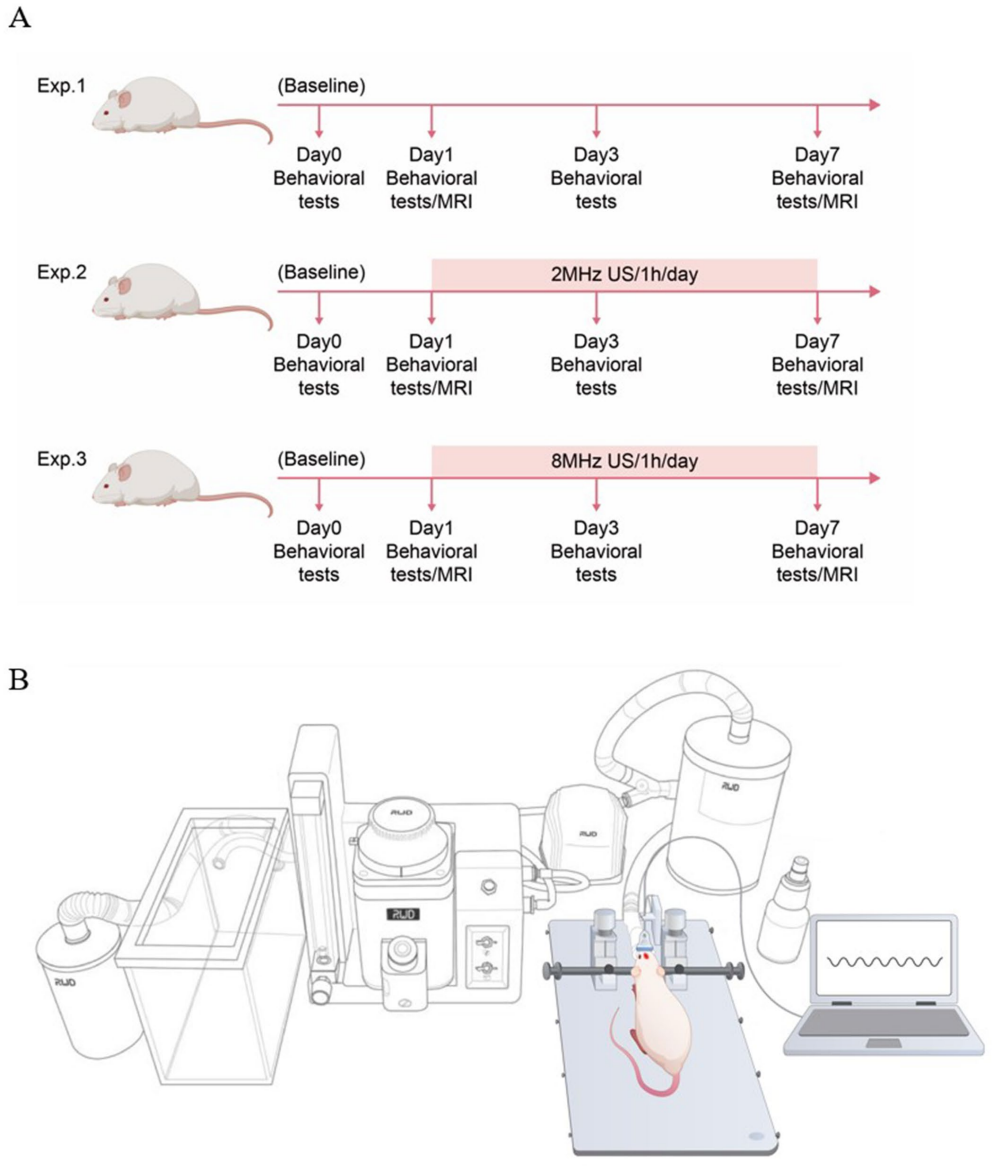
Experimental design. **(A)** Flow chart of the experimental procedure. Twenty-nine rats were randomized into control (ICH), ICH + 2 MHz ultrasound, and ICH + 8 MHz ultrasound groups. All animals were included in behavioral assessments conducted at baseline and at days 1, 3, and 7 post-ICH. A predefined longitudinal imaging subsample (*n* = 6 per group) was used for magnetic resonance imaging (MRI) analyses at days 1 and 7 post-ICH. **(B)** Schematic diagram of low-intensity pulsed ultrasound setup.

Animals were randomly assigned to treatment conditions. Randomization was conducted using the random number generator function in Microsoft Excel. A total of 29 rats were divided into three experimental conditions (Figure 1A). (Experiment 1) Rats received an intracerebral injection of 60 μL autologous whole blood into the right basal ganglia (*n* = 11). (Experiment 2) Rats received an infusion of 60 μL autologous whole blood and 2 MHz ultrasound treatment for 60 min per day over 7 consecutive days (*n* = 7). (Experiment 3) Rats received an infusion of 60 μL autologous whole blood and 8 MHz ultrasound treatment for 60 min per day over 7 consecutive days (*n* = 11).

All 29 animals were included in behavioral assessments, which were conducted at baseline (day 0) and at days 1, 3, and 7 post-ICH (*n* = 7 for the 2 MHz group, *n* = 11 for the ICH and 8 MHz groups). For magnetic resonance imaging (MRI) analyses, a predefined longitudinal imaging subsample was used. Six rats per group (*n* = 6/group) were randomly selected prior to imaging to undergo T2-weighted imaging (T2WI) and susceptibility-weighted imaging (SWI) at days 1 and 7 post-ICH. No animals were excluded from MRI analyses based on imaging outcomes.

### Pulsed ultrasound apparatus and exposure protocol

A non-invasive pulsed ultrasound system (Doppler-Box™, Compumedics Germany GmbH, DWL, Singen, Germany) equipped with dual-frequency probes (2 MHz and 8 MHz) was employed in this study. Although commercially labeled as a TCD device, the system was used exclusively as a source of therapeutic pulsed ultrasound exposure, rather than for diagnostic Doppler blood flow velocity measurements. No Doppler-specific functions, including spectral analysis, sample volume selection, gain adjustment, or flow velocity measurement, were applied.

Both probes were operated in pulsed-wave mode. Except for the spatial-peak temporal-average intensity (I_SPTA), which was set according to the system’s standard output settings, all other acoustic parameters were probe-inherent, manufacturer-defined presets and were not user-adjustable. Accordingly, these parameters were kept constant across all animals and experimental sessions and were not treated as experimental variables. For the 2-MHz probe, the maximum mechanical index (MI) was 0.5, the maximum thermal index was 2.8, I_SPTA was 420 mW/cm^2^, the peak negative acoustic pressure was 0.65 MPa, the pulse repetition frequency (PRF) was 7,000 Hz, and the pulse duration was 3.78 μs. For the 8-MHz probe, the maximum MI was 0.1, I_SPTA was 200 mW/cm^2^, the peak negative acoustic pressure was 0.41 MPa, the PRF was 7,000 Hz, and the pulse duration was 9.11 μs. All ultrasound exposures were performed within manufacturer-defined safety limits and in accordance with the ALARA (as low as reasonably achievable) principle.

Ultrasound coupling gel was applied to ensure appropriate acoustic impedance matching between the transducer and the rat skull. During ultrasound exposure and MRI procedures, rats were anesthetized with a 2% isoflurane/air mixture, and body temperature was maintained using a forced-air heating system.

The ultrasound transducer was stereotactically mounted (RWD Life Science, China) and positioned on the intact skull surface above the hematoma location, corresponding to the cranial burr hole used for ICH induction. Ultrasound was delivered transcranially to the underlying brain tissue. Because no Doppler sampling gate was used, parameters such as insonation depth selection and sample volume positioning are not applicable in this therapeutic context. Given the small skull thickness and limited scalp-to-brain distance in rats, transcranial ultrasound exposure from the cranial surface resulted in effective insonation of the cortical and perihematomal brain regions for both probe frequencies. Ultrasound treatment was administered once daily for 60 min/day over 7 consecutive days following ICH induction (Figure 1B).

### Magnetic resonance imaging

MRI was performed on a 7.0-T scanner (Bruker, Germany). During the MRI scans, the rats were anesthetized with intraperitoneal injection of pentobarbital (45 mg/kg). Their body temperature was maintained at approximately 37 °C. T2WI were acquired using a fat-suppressed RARE sequence (TR/TE = 3380/41 ms, field of view = 55 × 55 mm^2^, matrix size = 384 × 384, in-plane resolution = 0.143 × 0.143 mm^2^, slice thickness = 0.8 mm), with 27 consecutive coronal slices acquired to ensure full coverage of the hematoma, ventricular system, and surrounding brain tissue, for lesion volume assessment. SWI (TR/TE = 20/10 ms, field of view = 35 × 35 mm^2^, matrix size = 384 × 384, in-plane resolution = 0.091 × 0.091 mm^2^, slice thickness = 0.5 mm) was performed to quantify hematoma volume with 33 consecutive coronal slices acquired for complete lesion coverage. T2WI and SWI datasets were manually segmented using ITK-SNAP (version 3.8.0^1^), with hematoma and lesion volumes calculated by tracing regions of interest (ROIs). For quantitative analysis, ROIs were delineated on all consecutive slices covering the hematoma, ventricular system, and perihematomal region. Volumetric measurements were obtained by summing ROI areas across slices and multiplying by slice thickness. This volumetric analysis strategy applies to all MRI-based outcome measures, including parenchymal hematoma, intraventricular hematoma, intracerebral hematoma, and PHE. PHE volume was derived by subtracting hematoma volume from total lesion volume. MRI scans were conducted at days 1 and 7 post-ICH. In the present study, hematoma clearance rates were used as primary endpoint, which was calculated as: (hematoma volume at day 1- hematoma volume at day 7) hematoma volume at day 1 × 100%. In addition, PHE volume clearance rate was evaluated as well, which was calculated as:(PHE volume at day 1- PHE volume at day 7)/PHE volume at day 1 × 100%. All MRI analyses were conducted by two independent investigators blinded to group allocation. Each investigator independently analyzed the complete MRI dataset, including all animals and time points, using the same predefined ROI delineation protocol.

### Behavioral tests

The corner turn test and forelimb use asymmetry were used for neurological assessment as previously described (21). Both tests were performed at baseline (day 0) and post-ICH days 1, 3, and 7. Forelimb use asymmetry was quantified by recording the frequency of ipsilateral (I), contralateral (C), and bilateral (B) forelimb contacts during vertical rearing activity. The asymmetry score was calculated as: (I − C)/ (I + C + B) × 100%. In the corner turn test, rats freely entered a 30°-angled corner, and their turning direction (left/right) was recorded over 20 consecutive trials. The percentage of right turns relative to total trials was computed. All behavioral assessments were evaluated by an investigator blinded to experimental groups.

### Statistical analysis

Data were expressed as mean ± standard error of the mean (SEM). One-way and two-way ANOVA were employed to determine significant differences between groups. Bonferroni *post hoc* analysis was utilized for multiple comparisons correction. Statistical significance was defined as *p* < 0.05. All analyses were conducted using GraphPad Prism 9.0 (GraphPad Software, Boston, MA, United States).

## Results

### Ultrasound treatment and parenchymal hematoma clearance

**Figure 2:**
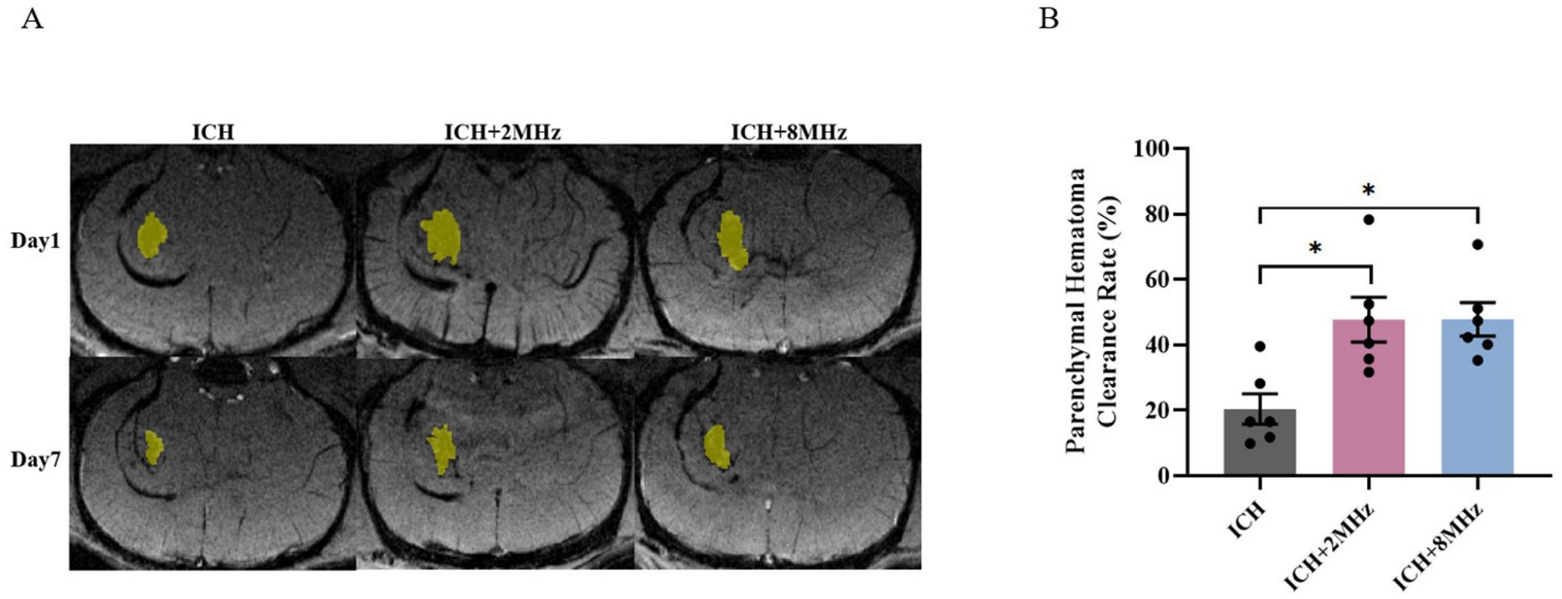
Ultrasound treatment enhances parenchymal hematoma clearance in a frequency-independent manner. **(A)** Representative SWI scans at days 1 and 7 post-ICH. **(B)** Quantification of hematoma clearance rates in control ICH, ICH + 2 MHz, and ICH + 8 MHz groups (*n* = 6/group). Individual data points represent single animals. Data expressed as mean ± SEM. *p < 0.05 vs. ICH group (one-way ANOVA with Bonferroni correction). Representative images are shown for visualization purposes, whereas quantitative analyses were based on multi-slice volumetric measurements.

Parenchymal hematoma size was evaluated using SWI at days 1 and 7 following intracerebral injection of 60 μL autologous arterial blood (Figure 2A). As shown in Figure 2B, ultrasound treatment significantly enhanced hematoma clearance rates in the ICH + 2 MHz group compared to untreated ICH controls (47.7 ± 6.86% vs. 20.40 ± 4.62%, *p* = 0.002). Similarly, the ICH + 8 MHz group also exhibited significantly higher hematoma clearance rates than untreated ICH controls (47.82 ± 5.10% vs. 20.40 ± 4.62%, *p* = 0.011). Notably, no intergroup difference was detected between the 2 MHz and 8 MHz ultrasound protocols (47.7 ± 6.86% vs. 47.82 ± 5.10%, *p* > 0.05).

### Ultrasound treatment and intraventricular hematoma clearance

**Figure 3:**
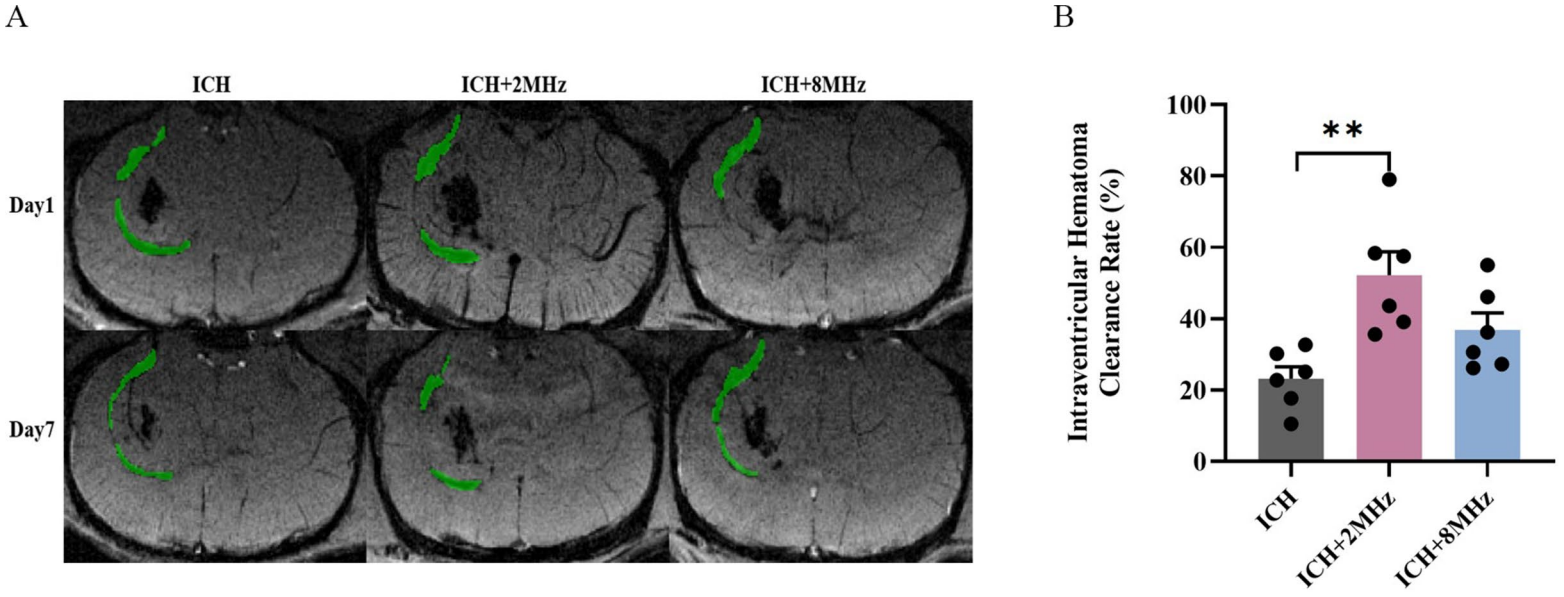
2 MHz ultrasound preferentially enhances intraventricular hemorrhage resolution. **(A)** Representative SWI scans demonstrating temporal changes in intraventricular hematoma volume. **(B)** Quantitative clearance rates stratified by ultrasound frequency (*n* = 6/group). Individual data points represent single animals. Data expressed as mean ± SEM. **p < 0.01 vs. ICH controls (one-way ANOVA with Bonferroni correction). Representative images are shown for visualization purposes, whereas quantitative analyses were based on multi-slice volumetric measurements.

Consistent with parenchymal hematoma analysis, intraventricular hematoma volume was serially assessed via SWI at days 1 and 7 post-ICH (Figure 3A). As shown in Figure 3B, the 2 MHz ultrasound group exhibited significantly enhanced intraventricular hematoma clearance compared to the control group (52.22 ± 6.60% vs. 23.17 ± 3.34%, *p* = 0.003). In contrast, the 8 MHz ultrasound group did not show a significant therapeutic effect on intraventricular hematoma clearance (36.9 ± 4.69% vs. 23.17 ± 3.34%, *p* > 0.05). Interprotocol comparison revealed no statistical difference between 2 MHz and 8 MHz regimens (52.22 ± 6.60% vs. 36.9 ± 4.69%, *p* > 0.05).

### Ultrasound treatment and intracerebral hematoma clearance

**Figure 4:**
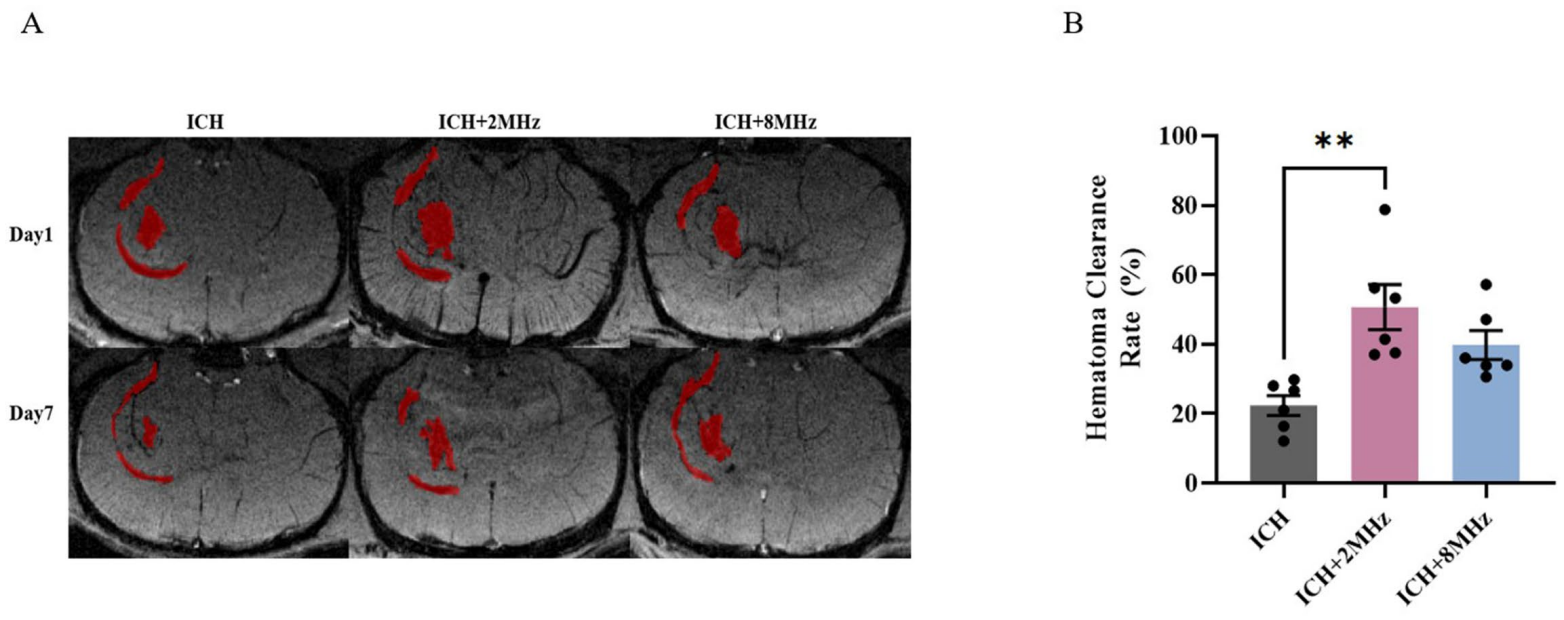
2 MHz ultrasound enhances intracerebral hemorrhage clearance. **(A)** Representative SWI scans showing hematoma dynamics. **(B)** Quantitative analysis of hemorrhage clearance rates (*n* = 6/group). Individual data points represent single animals. Data expressed as mean ± SEM. **p < 0.01 vs. ICH controls (one-way ANOVA with Bonferroni *post hoc* test). Representative images are shown for visualization purposes, whereas quantitative analyses were based on multi-slice volumetric measurements.

Intracerebral hemorrhage volume (parenchymal + intraventricular) was longitudinally monitored via SWI at days 1 and 7 post-ICH induction (Figure 4A). As shown in Figure 4B, the 2 MHz ultrasound group demonstrated significantly superior clearance efficacy compared to the control group (50.72 ± 6.51% vs. 22.36 ± 2.88%, *p* = 0.002). In contrast, the 8 MHz ultrasound group did not achieve statistical significance in clearance efficacy (39.80 ± 4.18% vs. 22.36 ± 2.88%, *p* > 0.05). Inter-frequency comparison showed no statistical difference between protocols (50.72 ± 6.51% vs. 39.80 ± 4.18%, *p* > 0.05).

### Ultrasound treatment and PHE

**Figure 5:**
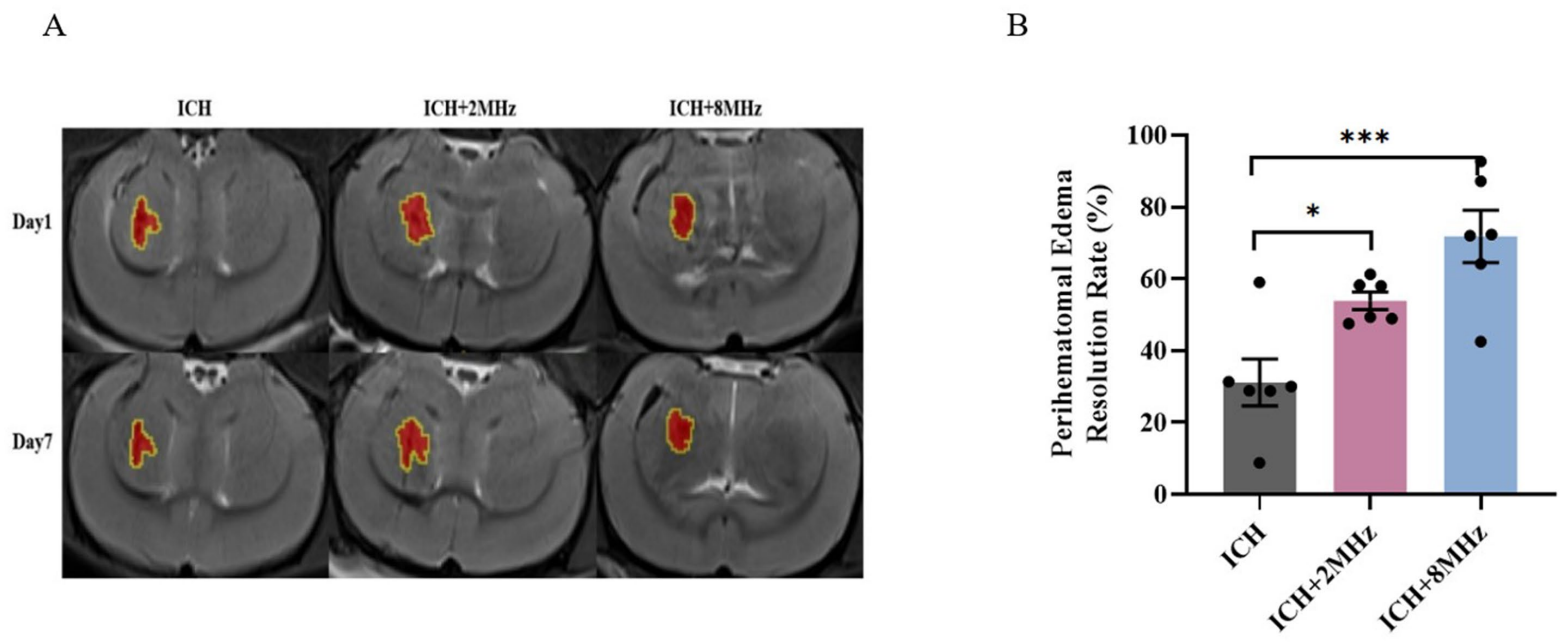
Frequency-specific ultrasound enhancement of PHE resolution**. (A)** Representative T2WI scans at days 1 and 7 post-ICH. *Red areas*: Parenchymal hematoma; *Yellow contours*: Total lesion boundaries. **(B)** Quantitative analysis of PHE volume changes (*n* = 6/group). Individual data points represent single animals. Data expressed as mean ± SEM. Statistical significance determined by one-way ANOVA with Bonferroni *post hoc* test (*p < 0.05, ***p < 0.001 vs. ICH controls). Representative images are shown for visualization purposes, whereas quantitative analyses were based on multi-slice volumetric measurements.

Lesion progression was longitudinally monitored via T2WI at days 1 and 7 post-ICH induction (Figure 5A). PHE volume was calculated as [Lesion volume – Parenchymal hematoma volume] (Figure 5A). As shown in Figure 5B ultrasound treatment significantly enhanced PHE resolution rate in the ICH + 2 MHz group compared to untreated ICH controls (53.91 ± 2.42% vs. 31.15 ± 6.55%, *p* = 0.043). Similarly, the ICH + 8 MHz group also exhibited significantly higher PHE resolution rate than untreated ICH controls (71.83 ± 7.28% vs. 31.15 ± 6.55%, *p* = 0.001). Inter-frequency comparison revealed no statistical significance (71.83 ± 7.28% vs. 53.91 ± 2.42%, *p* > 0.05).

### Ultrasound treatment and neurological deficits improvement

Neurological function was longitudinally assessed using corner turning and cylinder tests at baseline (pre-surgery) and at days 1, 3, 7 post-ICH to evaluate ultrasound-mediated functional recovery. ICH induction precipitated significant neurological deterioration (vs baseline, *p* < 0.001), which was attenuated by ultrasound treatment at day 7 (*p* < 0.05).

**Figure 6:**
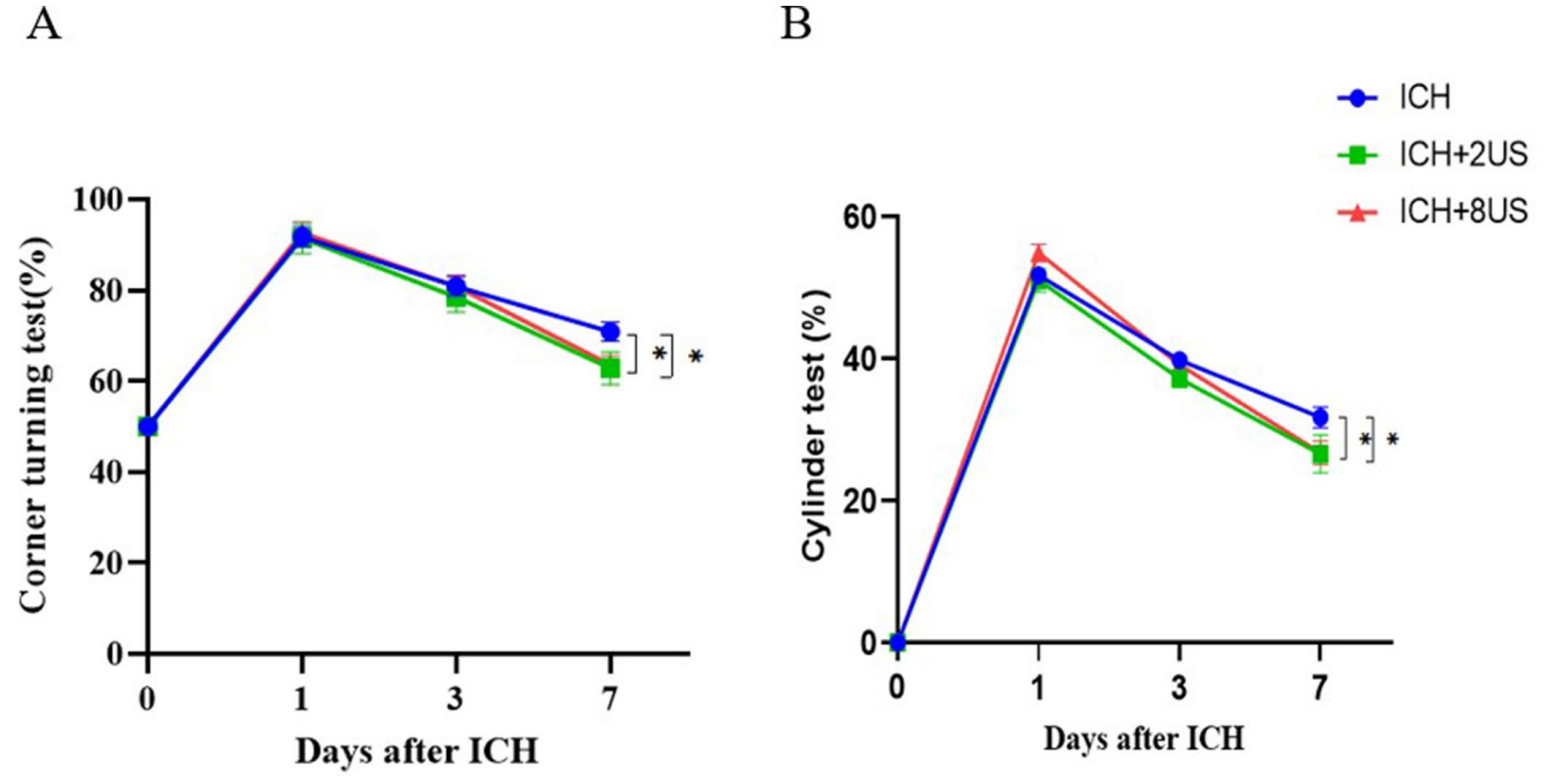
Ultrasound treatment rescues early neurological deficits post-ICH. **(A)** Corner test performance. **(B)** Cylinder test forelimb asymmetry scores. Data expressed as mean ± SEM (*n* = 7 for ICH + 2 MHz; *n* = 11 for ICH/ICH + 8 MHz). **p < 0.01, *p < 0.05 vs. ICH controls (two-way RM ANOVA with Bonferroni correction).

Corner test analysis demonstrated:2 MHz: 62.86 ± 3.60% vs. Control 70.91 ± 2.11% (*p* = 0.049); 8 MHz: 63.64 ± 2.03% vs. Control 70.91 ± 2.11% (*p* = 0.041) (Figure 6A). Cylinder test quantification revealed analogous improvements:2 MHz: 26.56 ± 2.72% vs. Control 31.75 ± 1.48% (*p* = 0.022); 8 MHz: 26.73 ± 1.65% vs. Control 31.75 ± 1.48% (*p* = 0.010) (Figure 6B). Interprotocol comparisons showed no frequency-dependent differences (*p* > 0.05).

## Discussion

In this study, we systematically evaluated the effects of non-invasive pulsed ultrasound on parenchymal hematoma clearance, PHE resolution, and neurological recovery in a rat model of intracerebral hemorrhage (ICH). Our findings demonstrate that: (1) Non-invasive pulsed ultrasound treatment significantly accelerated parenchymal hematoma clearance and effectively promoted the resolution of PHE; (2) Non-invasive pulsed ultrasound treatment significantly attenuated secondary brain injury following ICH, as evidenced by improved neurological function and reduced motor asymmetry in both the corner turn and cylinder tests (*p* < 0.05). These results highlight the translational potential of non-invasive pulsed ultrasound therapy for enhancing hematoma clearance, mitigating secondary injury, and improving functional outcomes after ICH.

Masomi-Bornwasser et al. previously investigated the thrombolytic effects of ultrasound in both *in vitro* and *in vivo* ICH models (22, 23). *In vitro* clot model, high-frequency ultrasound (10 MHz) significantly reduced relative clot weight (40.2% vs. 61%) even without pharmacologic agents, especially in large (50 mL) or aged (48-h) clots, underscoring the intrinsic thrombolytic capacity of ultrasound (22). In a porcine model, ultrasound-assisted drainage achieved an 18 ± 8% reduction in hematoma volume, superior to drainage alone (2 ± 1%) (23). These findings highlight the mechanical disruption of fibrin networks by ultrasound, though its standalone efficacy *in vivo* remains limited. Notably, the *in vitro* model lacks physiological complexity such as metabolism, inflammation, and blood–brain barrier integrity, while the *in vivo* porcine model, despite anatomical relevance, requires invasive procedures that may hinder clinical translation.

In another recent study, Su et al. demonstrated that LIPUS exerts anti-inflammatory and neuroprotective effects by modulating glia-mediated inflammation via the PI3K/Akt–NF-κB signaling pathway (24). Although this work provided valuable mechanistic insights, it primarily focused on molecular pathways rather than therapeutic outcomes.

Building upon these findings, our study represents a translational advancement by utilizing non-invasive pulsed ultrasound to assess therapeutic efficacy in ICH. Through stereotactic ultrasound delivery, longitudinal MRI-based hematoma quantification, and behavioral assessments, we demonstrated that non-invasive pulsed ultrasound significantly promotes hematoma clearance, reduces PHE, and improves functional recovery. This approach bridges the gap between mechanistic investigations and clinically relevant interventions, underscoring the potential of ultrasound as a non-invasive therapy for ICH.

Larger hematoma volumes are closely associated with poor neurological outcomes (25). Our study showed that non-invasive pulsed ultrasound significantly enhanced hematoma clearance (47.7 ± 6.86% vs. 20.40 ± 4.62%, *p* = 0.002). This is consistent with *in vitro* evidence demonstrating that ultrasound alone can achieve clot lysis efficiencies comparable to recombinant tissue plasminogen activator (rtPA) monotherapy (26). Mechanistically, ultrasound in the kHz–MHz range has been shown to synergistically enhance rtPA activity; for example, 1 MHz irradiation increased clot degradation rates by 1.8-fold compared to rtPA alone (27). Clinical studies have further supported this, showing that ultrasound-assisted thrombolysis in acute ischemic stroke improves 24-h outcomes (20). Our findings suggest that non-invasive pulsed ultrasound may be a promising therapeutic approach for ICH, potentially applicable in clinical contexts.

PHE is a critical pathological process that leads to delayed neurological deterioration after intracerebral hemorrhage (ICH), driven by thrombin activation, immune dysregulation, BBB dysfunction, and hemoglobin cytotoxicity (28–31). The rapid expansion of PHE exacerbates intracranial hypertension, imposing mechanical stress on brain tissue and causing neuronal injury (32). In our study, non-invasive pulsed ultrasound treatment facilitated the absorption of PHE. Previous studies have demonstrated that transcranial ultrasound–based stimulation, including transcranial focused ultrasound and LIPUS exerts neuroregulatory and neuroprotective effects across various neurological conditions (17, 33). In a middle cerebral artery occlusion mouse model, low-intensity, low-frequency (0.5 MHz) transcranial focused ultrasound applied at 2, 4, and 8 h after ischemia enhanced tight junction protein ZO-1 expression, reduced IgG leakage, improved blood–brain barrier integrity, and attenuated TNF-α secretion and MMP-9 activation, thereby reducing brain edema volume (33). Similarly, transcranial LIPUS demonstrated significant neuroprotective effects in a traumatic brain injury mouse model by alleviating brain edema, decreasing MMP-9 activity, mitigating neutrophil infiltration, and inhibiting microglial activation (17). These findings suggest that the neuroprotective effects of LIPUS are associated with its ability to mitigate early inflammatory events. Our results are consistent with these prior studies, indicating that non-invasive pulsed ultrasound may promote PHE resolution by modulating neuroprotective and anti-inflammatory pathways. However, further research is needed to elucidate the precise molecular mechanisms underlying these therapeutic effects.

An important aspect of the present study is the comparison between 2 MHz and 8 MHz pulsed ultrasound exposure. Despite their distinct acoustic properties, both frequencies significantly promoted parenchymal hematoma clearance, reduced perihematomal edema, and improved neurological function compared with untreated ICH controls. Notably, no marked superiority of one frequency over the other was observed with respect to parenchymal hematoma resolution, suggesting that, within the frequency range examined, the overall therapeutic effects of ultrasound are not exclusively determined by frequency alone. Instead, these benefits are likely mediated by shared mechanical and biological mechanisms, such as microstructural clot modulation, altered interstitial transport, and modulation of local inflammatory responses.

By comparison, differences between frequencies emerged in intraventricular hemorrhage resolution, where 2 MHz ultrasound demonstrated a more pronounced effect than 8 MHz. This finding may be related to the greater penetration depth and broader acoustic field associated with lower-frequency ultrasound, which could be advantageous for influencing deeper or ventricular components of hemorrhage. In contrast, the higher-frequency 8 MHz ultrasound may generate more spatially confined mechanical stimulation, which appears sufficient for parenchymal hematoma but less effective for intraventricular regions.

The mechanisms underlying ultrasound-mediated clearance of parenchymal hematoma and perihematomal edema are not fully understood. However, based on previous research, we can propose several potential modes of action: (1) Mechanical Effects: Ultrasound waves can fragment thrombi through vibrations, breaking them into smaller particles and accelerating their metabolic clearance (18). Additionally, acoustic streaming generates shear forces that disrupt thrombi (34). (2) Cavitation Effects: Microbubbles oscillate and implode under ultrasound, disrupting thrombi and promoting their dissolution (35, 36). (3) Hemodynamic Modulation: Ultrasound application is thought to improve hemodynamics by augmenting blood flow in brain tissue (37, 38). This augmentation of blood flow velocity and circulation facilitates the transportation of mononuclear phagocytes to the perilesional area, thus promoting more efficient hematoma clearance. (4) Cell Membrane Alterations: Ultrasound increases cell permeability, enhancing fluid and solute exchange, which may accelerate hematoma absorption (39, 40). (5) Ultrasound reduces inflammation by inhibiting inflammatory factor production, thereby reducing edema and improving the local microenvironment (32, 41). (6) Neuroprotective Effects: Studies have shown that ultrasound exerts neuroprotective effects on nerve cells (15). This potentially alleviates cellular stress and promotes neuronal survival, thus mitigating damage to the surrounding brain tissue caused by hematomas. These mechanisms likely interact synergistically to promote hematoma clearance, perihematomal edema resorption, and brain tissue repair. Future research should focus on elucidating the specific roles and interactions of these mechanisms to optimize therapeutic strategies.

This study has several limitations. First, the specific biological mechanisms through which non-invasive pulsed ultrasound mitigates ICH-induced damage remain to be clarified. Second, the findings are based on a single-center rodent model, which may not fully recapitulate human pathophysiology. Multicenter studies utilizing standardized protocols are needed to validate reproducibility. Finally, the translational relevance of our findings should be evaluated in large animal models, such as pigs, which offer more accurate anatomical and acoustic properties relevant to clinical application.

## Conclusion

Non-invasive pulsed ultrasound facilitated hematoma clearance, the resorption of perihematomal edema, and mitigated neuronal damage following ICH. Our findings demonstrate that non-invasive pulsed ultrasound treatment represents a potential therapeutic strategy for ICH.

## Data Availability

The raw data supporting the conclusions of this article will be made available by the authors, without undue reservation.

## Footnotes

1
http://www.itksnap.org
